# Evolving imaging methods of prostate cancer and the emergence of magnetic resonance imaging guided ablation techniques

**DOI:** 10.3389/fonc.2022.1043688

**Published:** 2022-11-17

**Authors:** Mikael Anttinen, Roberto Blanco Sequeiros, Peter J. Boström, Pekka Taimen

**Affiliations:** ^1^ Department of Urology, University of Turku and Turku University Hospital, Turku, Finland; ^2^ Department of Diagnostic Radiology, University of Turku and Turku University Hospital, Turku, Finland; ^3^ Institute of Biomedicine and FICAN West Cancer Centre, University of Turku, Turku, Finland; ^4^ Department of Pathology, Laboratory Division, Turku University Hospital, Turku, Finland

**Keywords:** ablation therapy, HIFU, high-intensity focused ultrasound, MRI, magnetic resonance imaging, Tulsa, transurethral ultrasound ablation, prostate cancer

## Abstract

Established therapies for prostate cancer (PCa), surgery and radiotherapy, treat the entire gland regardless of the location of the cancerous lesion within the prostate. Although effective, these methods include a significant risk of worsening genitourinary outcomes. Targeted image-guided cancer therapy has gained acceptance through improved PCa detection, localization, and characterization by magnetic resonance imaging (MRI). Minimally-invasive ablative techniques aim to achieve comparable oncological outcomes to radical treatment while preserving genitourinary function. Transurethral ultrasound ablation (TULSA) and next-generation transrectal high-intensity focused ultrasound (HIFU) utilize MRI guidance to thermally ablate prostate tissue under real-time MRI monitoring and active temperature feedback control. Previous trials performed by our group and others, including a large multicenter study in men with localized favorable-risk disease, have demonstrated that TULSA provides effective prostate ablation with a favorable safety profile and low impact on quality of life. Recently, MRI-guided HIFU focal therapy was also shown as a safe and effective treatment of intermediate-risk PCa. Here we review the current literature on ablative techniques in the treatment of localized PCa with a focus on TULSA and HIFU methods.

## Introduction

Prostate cancer (PCa) is the second most common cancer in men worldwide (1) and mainly a disease of older age groups. Due to the development of diagnostics and increased PCa awareness, the condition is diagnosed earlier and at a younger age (2). Moreover, the prostatic tumor affecting prognosis can be frequently visualized with newer imaging methods, becoming a target for imaging-guided cancer therapy.

Although PCa is the sixth leading cause of cancer-related death in men worldwide, the disease is frequently chronic in a large proportion, and many men die of reasons other than PCa (1, 3). In addition to the development of diagnostics and treatments, this is explained by the relatively slow natural course of PCa. The oncological benefits of local treatments with radical goals are visible after decades, while the genitourinary adverse effects typically appear shortly after these treatments (4). Given the relatively long PCa-specific survival, many radically treated men have to cope with treatment-related comorbidities for a long time (4).

At the time of diagnosis, many PCas are organ-confined with a favorable prognosis, lacking many of the characteristics typical of cancer; including the ability to grow beyond the prostate capsule or metastasize (5). These favorable risk cases can be safely monitored within surveillance protocols (6). More aggressive, Gleason grade ≥ 3 + 4 and ISUP (International Society of Urological Pathology) grade group (GG) ≥ 2 PCas are treated with curative goals if the patient’s life expectancy is long enough, 8-15 years, depending on the risk category. In addition to histopathology, the widely used risk classification of the European Association of Urology takes into account the level of prostate-specific antigen (PSA) and the local extent of PCa based on digital rectal examination of the prostate (6). Based on these variables, localized PCa is divided into a group of low (clinically insignificant), intermediate or high risk of recurrence after local radical intent therapy (6).

The established treatment methods for localized PCa, surgery and radiotherapy, are effective treatments but frequently cause significant long-term adverse effects on urinary, sexual, and bowel functions (7). In both treatment methods, the risk of recurrence increases according to the risk category of the disease. Local recurrence after surgery is treated with radiotherapy of the surgical bed, in some instances including pelvic lymph node regions, and with or without androgen deprivation therapy (ADT) (6). Local treatments for locally relapsing PCa after radiotherapy are more challenging. The available salvage treatments, surgery and additional radiotherapy, are associated with high risks of significant complications and quality of life harms concerning the benefits achieved. Therefore, in many cases, recurrence is first monitored and ADT is initiated as the disease progresses. In ADT, the serum testosterone is lowered to the castration level, or the effect of testosterone in the target tissue is prevented. The treatment is associated with significant deterioration of quality of life and an increased risk of cardiovascular problems (8). In some patients, the recurrence might be curable with local treatments and ADT could be postponed to the future. In addition to the toxicity of retreatments, the limited use of salvage treatments has partly been explained by the inability of traditional diagnostic methods to differentiate between local and metastatic recurrence reliably.

With new magnetic resonance imaging (MRI) methods and positron emission tomography-computed tomography (PET-CT), PCa detection and disease spread assessment has become more accurate. Recurrences can be located more reliably in PET-CT with PSMA (Prostate Specific Membrane Antigen) as a target, even with low PSA values. In this way, patients who might benefit from local re-treatments are found at an earlier stage. It has been hypothesized that new ablation treatment methods and focal therapy approaches can be used to treat primary localized PCa and locally recurrent PCa after radiotherapy radically, with less tissue damage, and improved quality of life. Preliminary scientific evidence has supported this assumption, and imaging-guided ablation treatments have gained approval and offer selected patients a potentially more optimal treatment option considering the benefit-risk ratio.

The purpose of this study is to review the current literature on ablation techniques for localized PCa with a particular focus on treatment methods that use therapeutic ultrasound and real-time MRI guidance for treatment delivery.

## Evolving imaging methods of PCa and rationale for focal therapy

In the conventional diagnostic pathway, the suspicion of PCa is based on an elevated PSA value and abnormal palpation of the prostate gland. The diagnosis is confirmed by histopathological examination of tissue samples taken from the prostate under ultrasound guidance. It is often not possible to reliably distinguish PCa with ultrasound. Therefore, 10-12-core template systematic biopsies are typically taken to cover the peripheral zone of the prostate where up to 80% of PCas originate. The challenge of traditional diagnostic tools, however, is insufficient accuracy in finding clinically significant PCa affecting the patient’s prognosis.

Over the last ten years, prostate MRI has revolutionized PCa diagnostics. High-quality prospective studies have demonstrated the ability of MRI and MRI-targeted biopsy to detect and exclude clinically significant PCa and to ignore clinically insignificant PCa (9). MRI has also refined the assessment of the local spread of PCa (9).

Bone scintigraphy and CT of the body are used to investigate the distant spread of PCa. The methods have low sensitivity and specificity in identifying metastases (10). Alongside traditional methods, PSMA PET-CT has emerged and taken the diagnostics from the macro to the molecular level. PSMA is a type II transmembrane glycoprotein, the amount of which increases in PCa depending on the aggressiveness (11, 12). A recent randomized study showed that PSMA PET-CT is a 27% more accurate method than bone scintigraphy and CT in the primary staging of men with high-risk PCa (10). PSMA PET-CT has also proven to be a promising method for prostate tumor identification and local spread assessment (13–16).

In primary localized clinically significant PCa, the standard is the treatment of the entire gland, regardless of the location of the cancerous tumor in the prostate. Established treatment methods include surgery (open, laparoscopic, robot-assisted radical prostatectomy), external beam radiotherapy, and brachytherapy (6). Even though surgery and radiotherapy techniques have evolved, they still carry risks of adverse effects on the urinary and genital organs and the bowel as the structures that maintain these functions in the vicinity of the prostate will be damaged during the treatments (4, 7). In more detail, compared with active monitoring, there is a significantly higher risk of sexual dysfunction (95%) and urinary incontinence (55%) six months after surgery, and of sexual (88%) and bowel dysfunction (5%) after radiotherapy (4). In a more contemporary population-based observational study (n=2005), up to half of patients treated with surgery or radiotherapy experienced severe erectile dysfunction and 10% suffered from long-term urinary incontinence (7). This has aroused interest in prostate tissue-sparing treatment and focal therapy, which aims to selectively eradicate clinically significant cancer to reduce the risk of metastasis without causing adverse effects that affect the quality of life (17).

A minority (15-30%) of PCa are unifocal and/or unilateral, confined to a specific part of the prostate, and traditionally considered suitable for focal therapy (18–20). In multifocal PCa, the cancer foci differ in aggressiveness (21–23). Evidence indicates that the prognosis is determined mainly by the so-called index tumor, which is typically the largest in size and harbors the highest grade (24–28). MRI and PSMA PET-CT reliably identify this index tumor (9, 13). Treating the most aggressive tumor in multifocal diseases might be sufficient to control the disease with fewer adverse effects (28, 29).

Although imaging methods for PCa have developed tremendously, the challenge of focal therapy is the accuracy of the imaging methods to identify all clinically significant cancer foci that shall be treated to minimize the risk of recurrence and metastatic spread of the disease. For example, MRI reliably identifies the index tumor but frequently misses smaller significant lesions in multifocal diseases (30–33). The comparison of preoperative MRI data and histopathology of radical prostatectomy specimens has also shown that MRI significantly underestimates tumor size; a margin of up to 12 mm is needed around the tumor determined in MRI for cancer to be radically treated histopathologically (34–36).

## Ablative techniques in the treatment of PCa

In recent decades, imaging-guided ablative treatment methods have been developed (37). In addition to the aforementioned limitations of cancer imaging, another challenge has been the accuracy of the energy delivery system. These two requirements are necessary to estimate precise treatment margins and to optimize the oncological and functional outcomes. Promisingly, combining MRI with newer generation ablation methods has improved the treatment’s accuracy.

In ablation treatments, tissue-destroying energy is delivered to the prostate gland without a surgical wound. Most ablative methods utilize thermal energy to ablate prostate tissue, typically heating prostate tissue with radiofrequency, laser, or therapeutic ultrasound (37). Most research evidence has been accumulated on older generation ultrasound-guided high-intensity focused ultrasound (HIFU), cryoablation, irreversible electroporation, focal laser ablation and photodynamic therapy. High and low dose rate brachytherapy (HDR, high dose rate; LDR, low dose rate) and stereotactic ablative radiotherapy (SABR) have also been used in focal therapy. Other ablative treatment options, such as laser interstitial thermotherapy, radiofrequency ablation, and prostatic artery embolization have also been utilized for the treatment of localized PCa, but they are in the early phase of evaluation with a limited amount of data available (37). In general, ablative methods are less invasive than surgery and seem to have a more favorable side effect profile compared to traditional treatments (4, 7, 37). In focal ablative therapy, the treatment-emergent harm is expected to decrease even more. In the medium term, the oncological efficacy of focal ablative therapy seems non-inferior when compared to whole-gland treatment (38, 39). However, the patient selection criteria and the protocols for post-operative surveillance after ablation therapy and focal therapy approach remain to be established and it is evident that novel imaging methods will play a key role.

## MRI-guided ultrasound ablation methods

**Figure 1:**
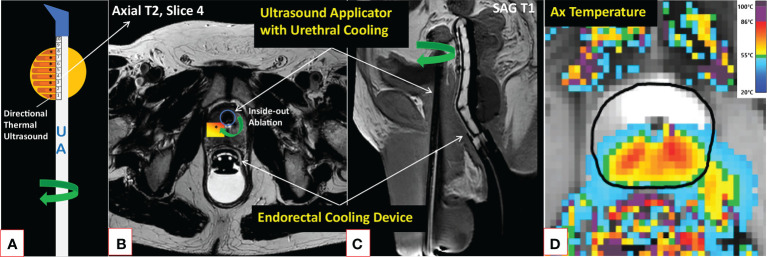
Description of the TULSA technology. MRI-guided TULSA is a minimally invasive ablation technique delivering directional high-intensity ultrasound energy (*) to the prostate (yellow circle) using a transurethral rotational UA comprised of 10 independently controlled ultrasound elements **(A)**. By actively cooling both the urethra and rectum throughout the ablation, TULSA protects these structures from thermal injuries **(B, C)**. Real-time MRI-thermometry is continuously acquired during the ablation to automatically control the delivered lethal thermal energy by adjusting each ultrasound element’s frequency and power and the UA’s rotation rate **(D)**. On the axial maximum temperature image of a patient undergoing lesion-targeted TULSA of a posterior peripheral zone tumor **(D)**, a minimum lethal temperature of 55°C reaches the drawn (black) boundary. Due to prostate swelling caused by the ablation, the catheter is kept in place for weeks after the procedure (see **Figure 2** patient case with a suprapubic catheter). MRI, magnetic resonance imaging; TULSA, transurethral ultrasound ablation; UA, ultrasound applicator.

**Figure 2:**
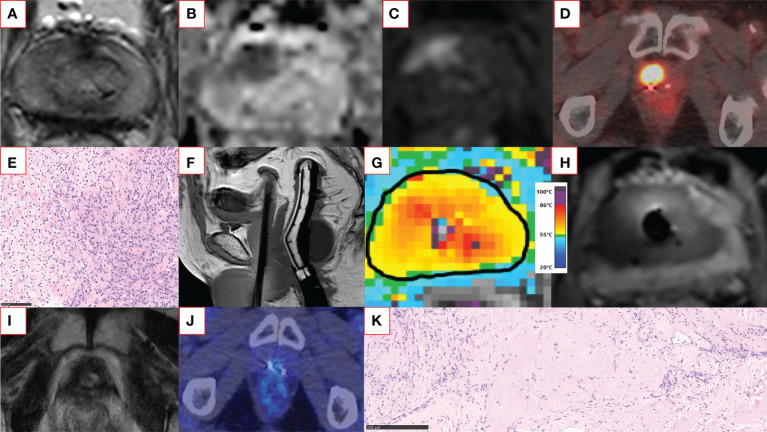
An example of a successful salvage TULSA patient case. The patient had a rising PSA of up to 13 ng/ml within six years after primary external beam radiotherapy. Screening T2-weighted **(A)** and diffusion-weighted **(B)** MRI showed a distinct anterior lesion with early enhancement on gadolinium-enhanced imaging **(C)** graded as PI-RR 5 lesion. The same lesion was also clearly visible in ^18^F-PSMA-1007 PET-CT (maximum standardized uptake value 23) **(D)**. Two residual gold fiducial markers implanted before image-guided radiotherapy are also visible next to the PSMA-positive lesion **(D)**. The MRI-targeted biopsy from the lesion revealed vital carcinoma resembling ISUP GG 5 disease **(E)**. The patient underwent whole-gland TULSA. On the sagittal T1-weighted image **(F)**, a transurethrally inserted ultrasound applicator, endorectal cooling device, and suprapubic catheter are in place. The targeted region reached a lethal minimum temperature of 55°C **(G)**. The non-perfused volume can be visualized immediately after treatment, demonstrating the acute ablation effect covering the targeted lesion **(H)**. At 12 months, the patient underwent follow-up imaging with multiparametric MRI and ^18^F-PSMA-1007 PET-CT **(I, J)**, both negative for cancer. The prostate volume decreased from 20 cm^3^ to less than 1 cm^3^ at 12 months. The 12-month post-TULSA biopsy agreed with imaging findings and showed only a treatment effect with no signs of cancer **(K)**. At the recent follow-up visit two years after TULSA, PSA is still low (PSA 0.067 ng/ml) and stable, and the patient has leak- and pad-free continence and erections sufficient for intercourse. The TULSA treatment report of the patient case, including treatment planning, thermal mapping, and post-treatment gadolinium-enhanced images, is provided in **Supplementary Figure S1**. CT, computed tomography; ISUP GG, International Society of Urological Pathology grade group; MRI, magnetic resonance imaging; PET, positron emission tomography; PI-RR, Prostate Imaging for Recurrence Reporting; PSA, prostate-specific antigen; PSMA, prostate-specific membrane antigen; TRUS, transrectal ultrasound; TULSA, Transurethral ultrasound ablation.

MRI-guided therapeutic ultrasound has been explored in the treatment of various benign and malignant solid tumors (40, 41). Recently, a novel MRI-guided transurethral ultrasound ablation (TULSA) (TULSA-PRO, Profound Medical Inc., Mississauga, Canada) has been evaluated in the treatment of various PCa conditions (42) (**Figure 1**). In contrast to a series of rapid small volume exposures in HIFU, directional ultrasound utilized in TULSA technology has distinct patterns of thermal dose and temperature deposition and subsequent tissue damage due to the use of continuous heating with an unfocused ultrasound beam (43). Both TULSA and HIFU performed inside the MRI scanner, known as magnetic resonance guided focused ultrasound (MRgFUS) (ExAblate; Insightec; Miami, FL, USA), exploit MRI guidance to thermally ablate prostate tissue under real-time MRI monitoring and active temperature feedback control. In addition to the MRI-thermometry derived thermal mapping, the treatment success can be assessed immediately post-treatment using gadolinium-enhanced images to visualize the acute perfusion defect caused by the treatment, denoted as non-perfused volume (NPV) (40, 43) (**Figure 2** and **Supplementary Figure S1**). Immediate NPV, however, underestimates the extent of thermal injury substantially since it cannot observe delayed thermal injury (43).

MRI-guided ultrasound ablation methods can be considered versatile, as they enable therapy for the entire prostate gland or more locally and in several different indications, such as palliation, salvage, and benign prostatic obstruction (42–46) (**Figures 1**, **2**). MRI-guided ultrasound ablation can often also be renewed and the therapy does not prevent surgery or radiotherapy later (37, 42, 43). The main contraindications for MRI-guided ultrasound ablation are the same as for MRI. In addition, the size of the prostate, calcifications, cysts, or post-radiation fiducial seeds may limit the successful implementation of the treatment. Limitation of MRI-guided ultrasound ablation methods include the relative complex technical requirements of the devices including the prolonged in-bore magnet time and MR-compatible anesthesia equipment, which in turn carry additional costs.

## Ablation therapy as the first-line treatment of localized PCa

The largest prospectively collected cohort from ablative therapy of localized PCa is for whole-gland ultrasound-guided HIFU. The medium-term results of the data set (n=1002) were published in 2014 (47). At eight years, 76% of low-risk, 63% of intermediate-risk, and 57% of high-risk patients were biochemically disease-free (PSA<nadir plus two ng/ml). At a ten-year follow-up, the cancer-specific survival rate was 97%, and metastases were found in 6%. The erectile function was preserved in half, and severe urinary incontinence occurred in 3-6% and urinary disorders in 6-35%, depending on whether older or newer ultrasound-guided HIFU technology was used. It is noteworthy that ADT was used to reduce the prostate size in 39% of patients, and 38% received two and 2% three HIFU treatments.

In Britain, high-quality, prospective cohort studies have been conducted on ablative methods in treating localized PCa. Failure-free survival (FFS) has been used as the primary endpoint, defined as survival without local or systemic treatments, metastases, or PCa death. Two ablation treatments were allowed to measure the effectiveness. A study published in 2018 reported the five-year PCa treatment results of focal HIFU (n=625) with five-year FFS of 88% (48). The corresponding result from whole-gland HIFU (n=754) was 70% (49). When taking the ISUP GG into account, FFS was 92% in GG 1 disease, 87% in GG 2-3 disease, and 59% in GG 4-5 disease (48). Metastases-free survival was 98%, cancer-specific survival 100%, and overall survival 99%. Complications included infections (~10%) and endoscopic procedures due to urinary disorders (~10%), while 30% of patients had to undergo endoscopic procedures in whole-gland HIFU. The rectourethral fistula was reported in two patients, similar to whole-gland HIFU. Urinary incontinence occurred in only 2% of patients, while in whole-gland HIFU, incontinence occurred in 12% of patients. Of note, in both studies ADT was used to downsize the prostate (48, 49).

Lately, Bründl et al. reported long-term oncological outcomes of 560 patients undergoing whole-gland ultrasound-guided HIFU with a median follow-up of 15 years and a range of up to 21 years. At 15 years, the cancer-specific and metastasis-free survival rates for low-, intermediate-, and high-risk patients were 95%, 89%, 65%, and 91%, 85%, and 58%, respectively (50). The corresponding percentages for salvage treatment-free survival were 67%, 52%, and 28%, respectively. Reddy et al. also recently reported longer-term oncological outcomes of 1379 patients undergoing focal ultrasound-guided HIFU with a median follow-up of 32 months (51). Seven-year FFS for low-, intermediate- and high-risk patients was 88%, 68%, and 65%, with 18% of patients undergoing repeat focal treatment and 7% undergoing salvage whole-gland treatment. At seven years, salvage whole-gland and systemic treatment-free, metastasis-free, cancer-specific, and overall survival rates were 75%, 100%, 100%, and 97%. Clavien Dindo adverse events greater than two occurred in 0.5% of patients.

In 2021, two retrospective studies compared the efficacy of focal therapy and traditional treatments in intermediate-risk PCa. Another study compared focal therapy (n=530, HIFU/cryotherapy/HDR-brachytherapy) with surgery (n=390) and radiotherapy (n=440) (52) and another compared focal therapy (n=246, HIFU/cryotherapy) with surgery alone (n= 246) (53). In both studies, the groups were compared using the propensity score matching method regarding significant prognostic factors. Neither study showed a demonstrable difference in efficacy (FFS) between the treatment groups at six and eight years. The only statistically significant difference was in the overall survival in favor of focal therapy. Of note, these two retrospective studies likely have included overlapping patient populations.

More recently, the results of a multicenter phase 2 study on the MRgFUS for patients with intermediate-risk PCa (n=101, 78% of patients with ISUP GG 2 disease) were published (54). Twenty-four-month biopsy outcomes demonstrated that the focal therapy with MRgFUS is a safe and effective treatment for ISUP GG 2 and 3 PCa with minimal deterioration of functional outcomes. Grade 1-2 urinary incontinence and erectile dysfunction occurred in 18% and 20% of patients. However, all patients reported pad-free continence and only a minor clinically insignificant decrease in functional erections by 24 months. At 24 months, 78 of 89 (88%) men had no evidence of ISUP GG 2 or higher PCa in the biopsies obtained from the treatment region and 59 of 98 (60%) men had no evidence of ISUP GG 2 or higher PCa anywhere in the prostate.

In a recent phase 2 multicenter study (TACT-trial), the efficacy of whole-gland TULSA treatment was demonstrated in low- and intermediate-risk PCa (n=115, 63% of patients with ISUP GG 2 disease) (55). At one-year biopsy , 72 of 111 (65%) men had no longer demonstrable cancer, and 14% had clinically insignificant disease (small volume ISUP 1 disease). Moreover, 52 of 68 (76%) men with ISUP GG 2 PCa at baseline were free of ISUP GG 2 disease anywhere in the prostate on 12-month biopsy. No severe complications occurred. Eight percent of patients had an infection or a urinary disorder as complications, 75% of patients maintained an erection sufficient for sexual intercourse without medication, and 96% of patients were continent at one year.

Safety and functional outcomes of the prospective and large retrospective studies evaluating ultrasound ablation of primary localized PCa are summarized in **Table 1**. The functional outcomes of these studies compare favorably to contemporary functional outcomes after surgery and radiotherapy (7). In a prospective, population-based cohort study from the United States including 1386 men with favorable-risk PCa, only 28% of men undergoing nerve-sparing radical prostatectomy and 51% of men undergoing external beam radiotherapy reported erections sufficient for penetration one year after treatment and 50% reported urinary incontinence requiring pad use one year after radical prostatectomy.

**Table 1:** Safety and functional outcomes of the studies evaluating ultrasound ablation of primary localized prostate cancer.

Author	Safety (Clavien Dindo or CTCAE when available)	Erections sufficient for penetration preserved at 12 months (%)	Pad-free continence preserved at 12 months (%)
Ahmed et al. (2012) (56)	Clavien grade 2: UTI 7/42, UR 1/42 Clavien grade 3: One patient underwent three endoscopic procedures for LUTS 1/42	89	100
Ahmed et al. (2015) (29)	Clavien grade 2: UTI 10/56 Clavien grade 3: Bladder neck incision 2/56, TURP 1/56	77	92
Feijoo et al. (2016) (57)	Clavien grade 2: UTI 4/71, UR 4/71 Clavien grade 3: TURP 2/71	52^1^	100^1^
Van Velthoven et al. (2016) (58)	Clavien grade 2: UTI 3/50, UR 4/50 Clavien grade 3: Endoscopic procedure for urinary stricture 2/50	80	94
Rischmann et al. (2017) (59)	Clavien grade 2: UTI 18 cases, UR 8 cases, phlebitis 2 cases, orchitis 8 cases, prostatitis 8 cases, urinary meatus stricture 1 case (Clavien grade 2-3), gross hematuria 5 cases (Clavien grade 2-3) Clavien grade 3: TURP 3/111	78	97
Guillaumier et al. (2018) (48)	Clavien grade 2: UTI 53/625 and/or epididymo-orchitis 12/625 Clavien grade 3: Rectourethral fistula 2/625, endoscopic procedure for LUTS 60/625	not reported	98^2^
Ganzer et al. (2018) (60)	Clavien grade 2: Urgency 2/51, UTI 9/51, UR 5/51 Clavien grade 3: 1/51 (internal urethrotomy for urethral stenosis)	70	96
Johnston et al. (2019) (61)	Clavien Grade 3: TURP 3/107, urethral stricture 2/107 (Clavien grade 2-3)	86	99
Abreu et al. (2020) (62)	Clavien grade 2: 5/100 UTI, UR 7/100 Clavien grade 3: 0	100^3^	100^3^
Nahar et al. (2020) (63)	Clavien grade 2: 13/52 Clavien grade 3: 4/52 (TURP for clot retention/necrotic tissue and for one patient concomitant drainage of scrotal abscess)	100	100
Shoji et al. (2020) (64)	CTCAE grade 2: 1/90 UTI CTCAE grade 3: 3/90 UTI, 3/90 urethral stricture	86	100
Ehdaie et al. (2022) (54)	Most common CTCAE Grade 1-2 events were hematuria 24/101 and UR 15/101 CTCAE Grade 3 (2%): UTI 1/101, urinary stricture 1/101 (treated with dilation)	84^4^	100^4^
Crouzet et al. (2014) (47)	UTI 39/1002, UR 76/1002, hematuria 55/1002, BOO 166/1002 and urinary strictures 90/1001 (treated with TURP or incision), urinary incontinence surgery 55/1002, rectourethral fistula 4/1002	42^5^	81^5^
Dickinson et al. (2016) (49)	UTI 58/754, recurrent UTI 22/754, epididymo-orchitis 22/754, endoscopic procedure 227/754, rectourethral fistula 1/754, osteitis pubis 1/754	39^6^	88^6^
Klotz et al. (2021) (55)	CTCAE grade 2: UTI (25%), epididymitis (5%), urethral stricture (1%) UR (9%), deep vein trombosis (1%) CTCAE grade 3: UTI (3%), epididymitis (1%), urethral stricture (2%), urethral calculus and pain (1%), urinoma (1%)	75	92

Studies with whole-gland ablation are in the grey rows. Urinary retention treated with Foley catheter was considered Clavien grade 2 adverse event.

BOO, bladder outlet obstruction; CTCAE, Common Terminology Criteria for Adverse Events; HIFU, high-intensity focused ultrasound; LUTS, lower urinary tract symptoms; MRgFUS, magnetic resonance guided focused ultrasound; TURP, transurethral resection of the prostate; UR, urinary retention; UTI, urinary tract infection.

^1^Assessed at 3 months after ultrasound-guided HIFU hemiablation.

^2^Assessed at 2-3 years after focal ultrasound-guided HIFU.

^3^Assessed within 2 years after ultrasound-guided HIFU hemiablation.

^4^Assessed at 2 years after focal MRgFUS.

^5^Assessed at 1-2 years after whole-gland ultrasound-guided HIFU.

^6^Assessed at last follow-up.

Focal and whole-gland prostate ablation treatment has been compared with traditional therapies in several systematic literature reviews and meta-analyses (37, 65, 66). However, without further prospective comparative, preferably randomized, studies, no firm conclusions can be drawn about the efficacy of ablation treatments relative to standard therapy. Most ablation studies have been done with small, single-center, and often retrospective datasets, where the follow-up periods have been short (**Table 2**). This might change shortly because, at least in the United States, a randomized multicenter study comparing TULSA treatment with surgery in intermediate-risk PCa is ongoing (NCT05027477).

**Table 2:** Prospective and largest retrospective studies evaluating ultrasound ablation of primary localized prostate cancer.

Author	Study design	No. of patients	Recruitment period	No. of centers	Intervention	With TURP (%)	Treatment strategy	Population ISUP GG	≥ISUP GG 2 (%)	Median follow-up (months)
Ahmed et al. (2012) (56)	Prospective	42	2007-2010	2	Ultrasound-guided HIFU	0	Focal	1-3	68	12
Ahmed et al. (2015) (29)	Prospective	56	2009-2011	1	Ultrasound-guided HIFU	0	Focal	1-3	65	12
Feijoo et al. (2016) (57)	Prospective	71	2009-2013	1	Ultrasound-guided HIFU	0	Focal (hemiablation)	1-2	13	12
Van Velthoven. (2016) (58)	Prospective	50	2007->	1	Ultrasound-guided HIFU	50 (50)	Focal (hemiablation)	1-3	40	35
Rischmann et al. (2017) (59)	Prospective	111	2009-2015	10	Ultrasound-guided HIFU	67 (60)	Focal (hemiablation)	1-2	26	30
Guillaumier et al. (2018) (48)	Prospective	625	2006-2015	9	Ultrasound-guided HIFU	NR*	Focal	1-5	72	56
Ganzer et al. (2018) (60)	Prospective	54	2013-2016	5	Ultrasound-guided HIFU	0	Focal (hemiablation)	1-2	16	17 (mean)
Stabile et al. (2019) (67)	Retrospective	1032	2005-2017	2	Ultrasound-guided HIFU	0	Focal	1-5	80	36
Johnston et al. (2019) (61)	Retrospective	107	NR	1	Ultrasound-guided HIFU	0	Focal	1-4	70	30
Abreu et al. (2020) (62)	Retrospective	100	2015-2019	2	Ultrasound-guided HIFU	11 (11)	Focal (hemiablation)	1-4	71	18
Nahar et al. (2020) (63)	Prospective	52	2016-2018	1	Ultrasound-guided HIFU	15 (29)	Focal	1-5	67	12
Shoji et al. (2020) (64)	Prospective	90	2016-2018	1	Ultrasound-guided HIFU	0	Focal	1-4	44	12
Ehdaie et al. (2022) (54)	Prospective	101	2017-2018	8	MRgFUS	0	Focal	2-3	100	24
Crouzet et al. (2014) (47)	Prospective	1002	1997-2009	1	Ultrasound-guided HIFU	939 (94)*	Whole-gland	1-5	45	77
Dickinson et al. (2016) (49)	Retrospective	569	2004-2012	8	Ultrasound-guided HIFU	NR*	Whole-gland	1-5	48	46
Klotz et al. (2021) (55)	Prospective	115	2016-2020	13	MRI-guided TULSA	0	Whole-gland	1-3	63	12

HIFU, high-intensity focused ultrasound; IDEAL, Idea; Development; Exploration; Assessment; Long-term; ISUP GG, International Society of Urological Pathology grade group; MRgFUS, magnetic resonance guided focused ultrasound; MRI, magnetic resonance imaging; NR, not reported; TULSA, transurethral ultrasound ablation.

*Use of androgen deprivation therapy prior to HIFU to downsize the prostate (Crouzet et al. 39% and Dickinson et al. 13%).

All prospective studies are single-arm studies. Early-phase studies (IDEAL stage <2b) are excluded. No randomized controlled trials have been published on ultrasound ablation of localized prostate cancer.

## Ablation therapy as a salvage treatment of radiorecurrent PCa

Up to 50% of PCa patients treated with radiotherapy will eventually present recurrence biochemically, which typically precedes clinical relapse (68). However, only a few receive salvage treatments due to their significant toxicity and limited cancer therapeutic efficacy (68). Part of the reason has been the paucity of different salvage treatment methods available. Surgery and additional radiotherapy have been available for a long time but only a few patients are eligible for salvage radical prostatectomy due to their general condition and comorbidities. In addition to these methods, most research evidence has been accumulated on ultrasound-guided HIFU and cryotherapy. A recent meta-analysis has compared the salvage methods listed above (69). Depending on the method, 61-100% of salvage treatments were given to the entire gland. Severe urogenital adverse effects occurred in 4-23% and intestinal in 0-2% of the patients. Toxicity seems to be the least significantly associated with HDR brachytherapy and SBRT; however, these methods were mostly used for focal therapy which may explain the differences. There was no significant difference in the therapeutic efficacy of cancer control between the salvage methods, and the effectiveness can be considered limited. Depending on the salvage method, 40-50% of patients presented disease recurrence during the five-year follow-up. More accurate patient selection with modern imaging methods may improve treatment outcome in the future and with newer ablation methods and focal treatment strategy, a better benefit-harm ratio may be achieved even in the salvage setting. In an early phase study from our group TULSA was shown to be safe and feasible treatment approach for whole-gland and focal ablation of radiorecurrent PCa with promising one-year oncological control (45) (**Figure 2** and **Supplementary Figure S1**). However, more studies with larger populations and longer follow-up are required to validate the efficacy of this treatment method in salvage indication.

## Conclusion

Ablative methods and focal treatment strategy seem to have a more favorable side effect profile than standard whole-gland treatments in primary localized and locally recurrent PCa after radiotherapy. In the medium term, focal therapy seems to be as effective as a treatment of the whole gland, at least in the primary treatment of intermediate-risk PCa. Recent observational cohort studies have shown no clinically or statistically significant difference in treatment response between radical and focal therapy. However, the scientific evidence for ablative methods and the usefulness of focal therapy is still limited, and the long-term efficacy has not been demonstrated. Randomized controlled studies are still needed to compare standard whole-gland treatments to ablative methods. In locally recurrent PCa after radiotherapy, with limited treatment options, ablation therapy offers a new treatment option in well-selected patients.

## Author contributions

MA: conceptualization, data curation, formal analysis, investigation, and writing—original draft. RBS: resources and investigation, funding acquisition, resources, supervision, writing—review and editing. PB: resources and investigation, funding acquisition, resources, supervision, writing—review and editing. PT: resources and investigation, funding acquisition, resources, supervision, writing—review and editing. All authors contributed to the article and approved the submitted version.

## Funding

PB reports grants from the Cancer Foundation Finland and the Hospital District of Southwest Finland. PT reports grants from the Cancer Foundation Finland and the Hospital District of Southwest Finland.

## Conflict of interest

MA reports grants from Profound Medical Inc, Finnish Urological Research Foundation, and Finnish Urological Association, and personal fees from Astellas, Bayer, Orion, and Janssen-Cilag, all outside the submitted work. PB reports personal fees from Profound Medical Inc and Janssen-Cilag Company outside the submitted work. PT reports personal fees from Roche, AstraZeneca, and MSD and non-financial support from MSD, all outside the submitted work.

The remaining author declares that the research was conducted in the absence of any commercial or financial relationships that could be construed as a potential conflict of interest.

## Publisher’s note

All claims expressed in this article are solely those of the authors and do not necessarily represent those of their affiliated organizations, or those of the publisher, the editors and the reviewers. Any product that may be evaluated in this article, or claim that may be made by its manufacturer, is not guaranteed or endorsed by the publisher.

## Supplementary material

Supplementary Figure 1 Intra-procedural MR images of the patient case (presented in **Figure 2**) undergoing salvage whole-gland TULSA for locally recurrent PCa after radiotherapy. On the top row, axial T2-weighted treatment planning images from all transducer elements (E1-E10) of the transurethral UA. The drawn yellow boundary on the prostate capsule (E3-E7) displays the target ablation area. On the second and third rows, maximum temperature and thermal dose images show a lethal minimum temperature of 55°C and a thermal dose of 240 CEM at 43°C covering the target area. On the fourth row, post-treatment contrast-enhanced images show the non-perfused volume with the rim of enhancement covering the target area. CEM, cumulative equivalent minute; MR, magnetic resonance; PCa, prostate cancer; TULSA, transurethral ultrasound ablation; UA, ultrasound applicator.
