# Supplementary figures and images for: Evolving imaging methods of prostate cancer and the emergence of magnetic resonance imaging guided ablation techniques
Source: Front Oncol. 2022 Nov 17;12:1043688. doi: 10.3389/fonc.2022.1043688 (PMC9714456; doi:10.3389/fonc.2022.1043688)

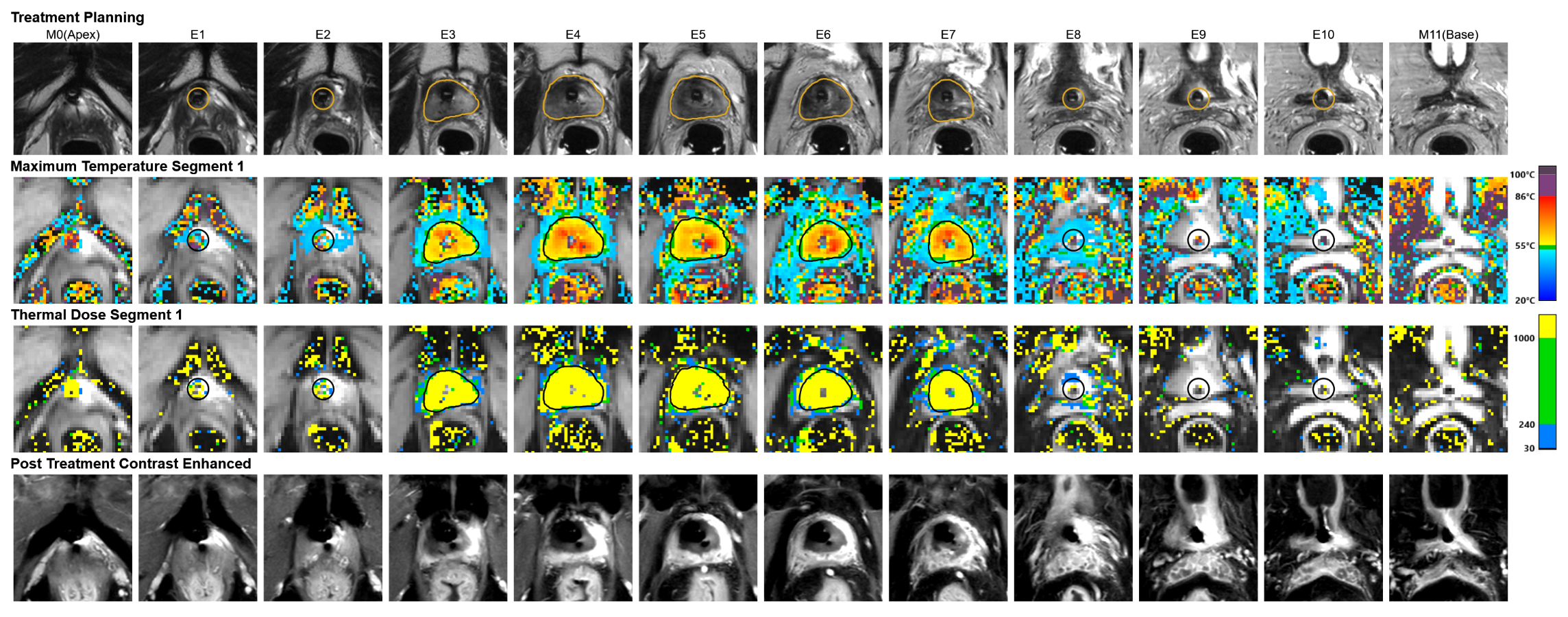

Supplement: Supplementary Figure 1 — Intra-procedural MR images of the patient case (presented in Figure 2) undergoing salvage whole-gland TULSA for locally recurrent PCa after radiotherapy. On the top row, axial T2-weighted treatment planning images from all transducer elements (E1-E10) of the transurethral UA. The drawn yellow boundary on the prostate capsule (E3-E7) displays the target ablation area. On the second and third rows, maximum temperature and thermal dose images show a lethal minimum temperature of 55°C and a thermal dose of 240 CEM at 43°C covering the target area. On the fourth row, post-treatment contrast-enhanced images show the non-perfused volume with the rim of enhancement covering the target area. CEM, cumulative equivalent minute; MR, magnetic resonance; PCa, prostate cancer; TULSA, transurethral ultrasound ablation; UA, ultrasound applicator. [file Image_1.tif]
